# Construction of Enterprise Financial Information Intelligent Processing Innovation Model Based on Internet of Things Technology

**DOI:** 10.1155/2022/7153260

**Published:** 2022-05-06

**Authors:** Mei Ding

**Affiliations:** Zhushan College, Qingdao Huanghai University, Qingdao, Shandong 266427, China

## Abstract

The application of Internet of Things technology provides conditions for the systematization of enterprise financial data and the intellectualization of financial management. In this study, support vector machine (SVM) algorithm and genetic algorithm (GA) are combined to obtain the innovation model of enterprise financial information intelligent processing based on GA-SVM optimization algorithm. Nine factors affecting enterprise financial information processing from 2010 to 2018 are selected as influencing factors, and according to the idea and method of data modeling, the simulation experiment of enterprise financial information intelligent processing model is carried out with MATLAB. The results show that the average fitness of GA-SVM is close to the best fitness, indicating that each individual in the population is near the optimal solution. The RMSE of GA-SVM is 0.35%, and the root mean square error obtained is greater than this value, which shows that the application effect of GA-SVM is significantly better than that of SVM using cross validation method, and it is more suitable for precision prediction. At the same time, it also reflects that the optimization of SVM parameters by GA is reliable. The algorithm can be better applied in the intelligent processing of enterprise financial information and can provide a certain reference for enterprises in the financial management based on the Internet of Things.

## 1. Introduction

With the development trend of China's market economy in full swing, the Internet of Things technology has developed rapidly and has become one of the key technologies applied by various enterprises [1]. The development of enterprises is inseparable from advanced technology, especially the financial management of enterprises [2]. In the era of Internet of Things, enterprise financial information management has become the key to enterprise development. Enterprises must update traditional concepts and make full use of advanced Internet of Things technology in order to effectively complete financial management [3]. The Internet of Things is a system based on the Internet, which realizes the connection between things and realizes monitoring at any time through modern radio frequency identification, infrared induction, and wireless communication technologies [4]. Internet of Things technology is widely used in major industries, which can effectively supervise enterprise financial information, realize the whole process management of enterprise financial information, make enterprise financial information processing more efficient, timely, and reliable, and finally improve the efficiency of enterprise financial management [5].

For the innovation model of enterprise financial information intelligent processing, this research combines the related technologies of genetic algorithm (GA) and support vector machine (SVM), introduces the genetic algorithm GA into the SVM parameters of support vector machine, and constructs the innovation model of enterprise financial information intelligent processing according to the idea and method of data modeling. Finally, MATLAB is used for simulation experiment.

This study mainly adopts the combination of research methods and simulation experiments, takes the idea of system engineering and complexity theory as the direction, extracts the data affecting enterprise financial information processing from 2010 to 2018, adopts the method of normalized data, and constructs the innovation model of enterprise financial information intelligent processing according to the idea and method of data modeling, The purpose is to improve the operation speed of the model and ensure the accuracy of the system.

The research content of this paper mainly includes four parts. The second part is the research status of genetic algorithm (GA-SVM) for support vector machine optimization at home and abroad. The third part mainly introduces the research algorithm. The first section describes the design of GA-SVM algorithm, and the second section discusses the implementation of GA-SVM algorithm. The fourth part preprocesses the influencing factors, empirically analyzes the GA-SVM algorithm, and compares the results with the traditional SVM algorithm. The results show that the application effect of GA-SVM is significantly better than that of SVM using cross validation method, and it is more suitable for precision prediction.

## 2. Related Works

In recent years, Internet of Things technology has been applied more and more in enterprise financial information processing, and researchers at home and abroad have also conducted in-depth research on this technology. Luthra et al. used grey relational analysis (GRA) and analytic hierarchy process (AHP) to analyze the challenges faced by the adoption and diffusion of the Internet of Things in India, so as to help practitioners and decision makers remove the obstacles to the successful adoption and promotion of the Internet of Things [6]. Wang R and others designed a new inventory pledge financing mode according to the special functions of Internet of Things technology and the business process of inventory financing mode. The results show that the risk loss value gap caused by various loss events in the Internet of Things combined with supply chain financial inventory pledge financing mode is large, among which the external fraud loss is the largest. Finally, it is found that the supply chain financing mode based on Internet of Things technology effectively reduces the operational risk [7]. Lopez BS and others analyzed the impact of artificial intelligence and Internet of Things technology on the change of business process management system. The Internet of Things allows you to deliver information, improve control and automation, and provide opportunities to optimize your company's operating costs [8]. Wen C. and others use distributed search engine technology to customize the web crawler to obtain the required bank card and transaction data from the multisource heterogeneous data of the Internet of Things financial industry, design the corresponding spark parallel algorithm to preprocess the data, and establish the inverted table and secondary index document, which provides a data source for the big data analysis platform. The results show that this method can significantly reduce the probability of banks formulating the first and second error rates and effectively reduce the losses caused by improper credit regulations when evaluating the credit risk of Internet of Things financial financing [9].

Genetic support vector machine algorithm (GA-SVM) has been highly valued by many relevant professionals. Dayma A. et al. proposed a content-based image retrieval system based on genetic support vector machine algorithm (GA-SVM). Users retrieve images by sending query images, extract visual features to retrieve query images, and implement it in MATLAB environment to verify the superiority of performance [10]. Yang et al. used GA-SVM to analyze the dissolved oxygen fault of water quality monitoring system. The results show that the optimized values of penalty coefficient and parameters after iteration are 2.1649 and 5.3312, respectively, and GA-SVM has good accuracy [11]. Yin et al. proposed a symbol detection method based on GA-SVM to deal with this problem from different angles and transformed the symbol decoding process into a numerical calculation process. The results show that compared with the traditional method of calculating threshold decoding symbols, GA-SVM improves the bit error rate (BER) performance of CBWCS, simplifies the symbol detection process, and eliminates the process of channel identification and threshold calculation [12]. Zhaia et al. proposed a hybrid method combining genetic algorithm (GA) and support vector machine (SVM). Genetic algorithm is used to mine key factors, and support vector machine is used to calculate the fitness function of genetic algorithm. Using the survey data of China Aviation Industry Corporation (AVIC), the effectiveness of the proposed hybrid method is experimentally analyzed. The experimental results show that the hybrid genetic support vector machine method proposed in this paper can be used as an alternative to the exploration of key factors [13]. Through the research of domestic and foreign scholars on genetic algorithm and support vector machine, it can be seen that the research method of combining genetic algorithm and support variable machine is the main research direction in the future. Therefore, this study mainly discusses the establishment of enterprise financial information intelligent processing innovation model, uses GA-SVM model algorithm to evaluate the processing of enterprise financial information, and uses MATLAB software to simulate and verify the algorithm.

## 3. Intelligent Financial Information Processing Model Based on GA-SVM Optimization Algorithm

### 3.1. GA-SVM Optimization Algorithm

**Figure 1:**
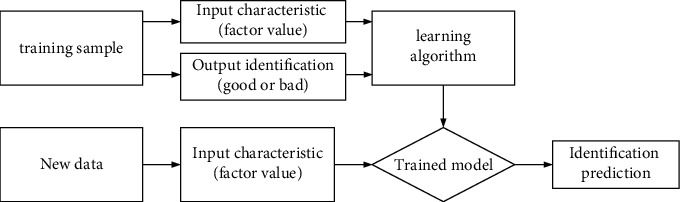
Traditional SVM design process.

Support vector machine can find the best scheme between learning ability and complexity according to the model with limited sample information, so as to obtain the minimum confidence range and empirical risk and obtain better generalization ability and statistical law when the statistical samples are incomplete [14]. Support vector machine (SVM) is a two-class classification model. Its basic model is the linear classifier with the largest interval defined in the feature space. Support vector machine also includes kernel technique, which makes it an essentially nonlinear classifier. The learning strategy of support vector machine is interval maximization, which can be formalized as a problem of solving convex quadratic programming, which is also equivalent to the minimization of regularized hinge loss function. The learning algorithm of support vector machine is an optimization algorithm for solving convex quadratic programming. The training of SVM needs to train the optimization of a quadratic programming. If the data capacity is large, it will cause a very large amount of computation of SVM [15]. If the training data is simply reduced, it will not only have a great impact on the accuracy and training effect of the classifier, but also affect the selection of parameters. The index selection of kernel function and penalty function also affects the prediction accuracy and model classification. Generally, the parameter indexes are continuously selected artificially and through repeated experiments, but human experience will cause certain subjectivity of the experiment and high time cost. The traditional support vector machine design process [16] is shown in Figure 1.

**Figure 2:**
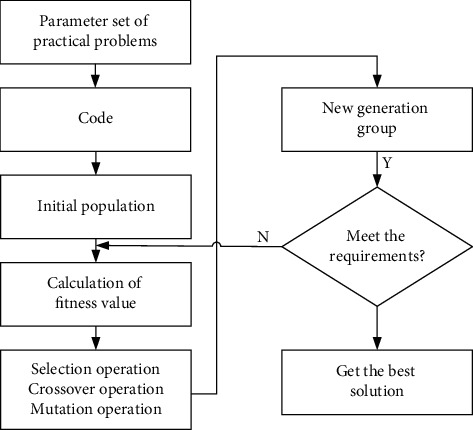
Implementation steps of genetic algorithm.

In this study, the parameter selection and kernel function of SVM are optimized by GA. The potential parallelism and global optimality of GA are not available in traditional algorithms. The implementation steps of GA are shown in Figure 2 [17]. This study uses the advantages of GA, proposes an improved method of SVM algorithm, and establishes a new GA-SVM algorithm model. The kernel function of GA-SVM takes the radial basis function, the model parameters are globally optimized and genetically coded by the real number coding method, and the final model parameters of GA-SVM adopt the searched optimal kernel parameter and penalty parameter C [18].

The specific ideas of GA-SVM model design are as follows: firstly, encode the parameters of SVM model. The process of finding the optimal kernel parameter and penalty parameter C is a complex parameter continuous optimization problem. In this study, the real number coding method is adopted to avoid repeated coding and decoding in the operation process. It can also solve the problem of limited binary coding length, so as to improve the accuracy and performance of genetic algorithm. If the penalty parameter C is large enough, it will lead to “over learning” of the model algorithm. At this time, the SVM model assigns all training samples to categories with large sample size.

SVM, which is based on the principle of structural risk minimization and statistical theory, is selected as a new machine learning method to effectively regress and classify nonlinear and linear data. SVM algorithm includes linear and nonlinear algorithms. When the data in the data set is linearly separable or approximately linearly separable, it maximizes a linear classifier through hard interval or soft interval. When the data set has a nonlinear structure, the nonlinear SVM is learned by maximizing the soft interval and using the kernel technique. For example, given a two-dimensional plane,

(1) $$\begin{array}{c} T=\left\{\left(x_{1},y_{1}\right),\left(x_{2},y_{2}\right),\ldots ,\left(x_{N},y_{N}\right)\right\}. \end{array}$$

In (1), *x*_*i*_ ∈ *R*^*n*^, *y*_*i*_{+1, −1},  *i*=1,2,…, *N* and *x*_*i*_ represent eigenvectors, *y*_*i*_ is the class mark of *x*_*i*_, and (*x*_*i*_, *y*_*i*_) represents sample points. Thus, it is extended to the n-dimensional hyperplane, where B represents the scalar and W represents the weight vector. By adjusting the scalar and weight vector, SVM searches the hyperplane on the side of the largest edge, as follows:

(2) $$\begin{array}{c} H_{1}:W\cdot X+b\geq 1,y_{i}=+1,\\ H_{2}:W\cdot X+b\leq 1,y_{i}=-1. \end{array}$$

The sample satisfying the above formula on the hyperplane *H*_1_ or *H*_2_ is the support vector. It is necessary to maximize the spacing and support the hyperplane, so it can be optimized twice, as follows:

(3) $$\begin{array}{c} \underset{W,b}{\min}\frac{1}{2}{\left\|W\right\|}^{2}, \end{array}$$

(4) $$\begin{array}{c} y_{i}\left(W\cdot x_{i}+b\right)\geq 1,\;i=1,2,\ldots ,N. \end{array}$$

The optimal solutions *W*^*∗*^ and *b*^*∗*^ can be obtained from (4), and the linear separable SVM and separation hyperplane can be obtained, as follows:

(5) $$\begin{array}{c} W^{*}\cdot x+b^{*}=0. \end{array}$$

Separate the decision function, as follows:

(6) $$\begin{array}{c} f\left(x\right)=\text{sign}\left(W^{*}\cdot x+b^{*}\right). \end{array}$$

If the decision data set is not linearly separable, there are two cases: linear approximate separability and nonlinear. When the data is linearly separable, the relaxation variable *ξ*_*i*_ needs to be introduced to make it reach the “separable” state, and the quadratic optimization representation is shown as follows:

(7) $$\begin{array}{c} \underset{W,b,\xi }{\min}\frac{1}{2}{\left\|W\right\|}^{2}+C\sum_{i=1}^{N}\xi i, \end{array}$$

(8) $$\begin{array}{c} y_{i}\left(W\cdot x_{i}+b\right)\geq 1-\xi ,\;i=1,2,\ldots ,N. \end{array}$$

Among them, *ξ*_*i*_ ≥ 0,  *i*=1,2,…, *N*. The optimal solution is obtained. The separation hyperplane and the separation decision function are shown in the following equations:

(9) $$\begin{array}{c} W^{*}\cdot x+b^{*}=0, \end{array}$$

(10) $$\begin{array}{c} f\left(x\right)=\text{sign}\left(W^{*}\cdot x+b^{*}\right). \end{array}$$

For the nonlinear data set, it needs to be transformed into a linear problem in the high-dimensional feature space for solution. The kernel function is used to replace the instance inner product in the nonlinear classification, the kernel function *K*(*x*, *z*)=*ϕ*(*x*) · *ϕ*(*z*). Accordingly, the obtained nonlinear SVM is shown in the following equation:

(11) $$\begin{array}{c} f\left(x\right)=\text{sign}\left(\sum_{i=1}^{N}\alpha_{i}^{*}y_{i}K\left(x,x_{i}\right)+b^{*}\right). \end{array}$$

The radial basis kernel function (RBF) is shown in the following equation:

(12) $$\begin{array}{c} K\left(x,x^{\prime }\right)=\exp\left(\frac{{\left\|x-x^{\prime }\right\|}^{2}}{2\sigma^{2}}\right). \end{array}$$

In (12), *K*(*x*, *x*′) represents the radial basis kernel function, *x* and *x*′ are two training points, and *σ* represents the kernel function, that is, the width in the function direction, the mean square deviation of the Gaussian function, and the *σ* value directly proportional to the function width.

Through the experimental verification of RBF kernel function, it can be seen that the value of *σ* has a close correlation with ‖*x* − *x*′‖^2^. If the minimum distance between training samples is much larger than *σ*, *σ*⟶0. If the minimum distance between training samples is much smaller than the *σ* value, then *σ*⟶*∞*. This study determines the search space for *σ* value, with the interval value being [min(‖*x* − *x*_*j*_‖^2^ × 10^−2^), max(‖*x* − *x*_*j*_‖^2^ × 10^−2^)], and further reduces the search range in this interval to determine a more accurate range.

Penalty coefficient C is mainly used to constrain Lagrange factor A. When the value of penalty coefficient C reaches a certain value, the constraint on *a* will fail. At this time, the complexity of SVM in data subspace will become the largest, and its generalization ability and empirical risk will not be changed. In this study, a new search method of c-interval is proposed. It can be seen from 0 ≤ *a*_*l*_, *a*_*i*_^*∗*^ ≤ *C*/*l*;  *i*=1,2,……, *l* that if *C* ≥ 0, first select a large enough C, and then apply the SVM model of this value to solve *a*_*i*_ (*i*=1,2,……, *n*), where *n* represents the number of training samples. At this time, if *C*_1_=max(*a*_*i*_) and *C*_1_ < *C*, then the upper bound of *C*'s search boundary is *C*_1_, indicating that *a*_*i*_ is still constrained by C. At this time, a larger capacity C should be selected to support SVM model training until C is much greater than *C*_1_. Therefore, the interval of C search can be confirmed, and the interval is (0, *C*_1_).

Then, the fitness function of genetic algorithm is designed. According to the experimental object of this study, a genetic fitness function is proposed as follows:

(13) $$\begin{array}{c} F\left(\sigma ,C\right)=\frac{1}{\text{Error}}. \end{array}$$

In (13), error represents the misclassification rate of the training sample set of SVM model. When the classification error of SVM model on the test sample is higher, the parameter chromosome fitness is smaller. Then, there is genetic operation, which includes selection operation, crossover operation, and mutation operation. The sorting of individuals in the population of the selection operation is carried out according to the fitness, and then the selected probability *P*_*i*_ of individuals is calculated, as shown in the following equation:

(14) $$\begin{array}{c} P_{i}=r\times {\left(1-r\right)}^{i-1}. \end{array}$$

In (14), *r* represents the selection probability of the first individual, and *i* represents the individual sorting sequence number. The selection probability has nothing to do with the fitness value, but only with the ranking of individuals in the population. Cross operation is a cross operation mode, which mainly applies the linear combination mode. If *x*_1_ and *x*_2_ chromosomes are crossed, it is shown in the following formula:

(15) $$\begin{array}{c} \left\{\begin{array}{c} x_{1}=ax_{1}+\left(1-a\right)x_{2},\\ x_{2}=ax_{2}+\left(1-a\right)x_{2}. \end{array}\right) \end{array}$$

In (15), *a* represents a random number and [0,1]. Mutation operation is to randomly select one of the mutation bits *j* in the mutated chromosome and transform the mutation bit *j* into a random number *U*(*a*_*i*_, *b*_*i*_). *a*_*i*_ represents the upper limit of the variation bit, and *b*_*i*_ represents the lower limit of the variation bit, as shown in the following equation:

(16) $$\begin{array}{c} x_{j}=\left\{\begin{array}{c} \begin{array}{cc} U\left(a_{i},b_{j}\right), & \left(i=j\right), \end{array}\\ \begin{array}{cc} x_{i}, & \left(i\neq j\right). \end{array} \end{array}\right) \end{array}$$

### 3.2. Construction of Intelligent Processing Model of Enterprise Financial Information

**Figure 3:**
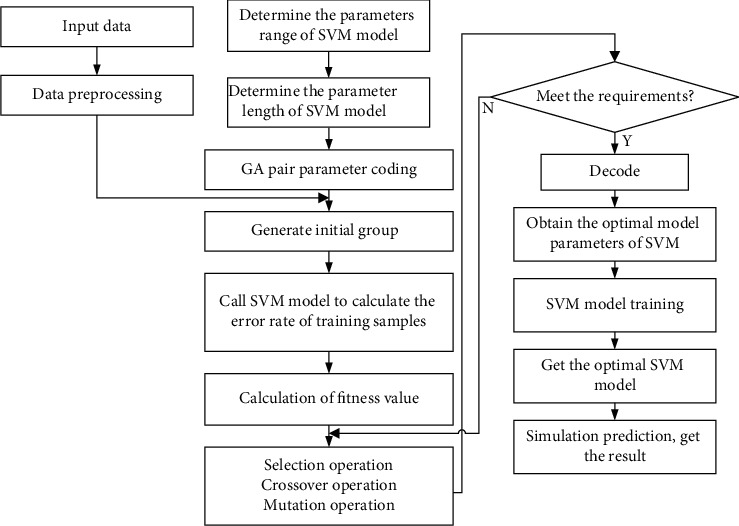
Implementation steps of GA-SVM algorithm.

After designing the GA-SVM algorithm, this study proposes an enterprise financial information intelligent processing model based on GA-SVM algorithm. The specific implementation steps are shown in Figure 3. Firstly, the regression model of support vector is designed to determine the values of kernel parameters and penalty coefficients. The individual length of GA is determined, *m* chromosomes are randomly generated by real number coding, and the initial population *P*(*t*) of genetic algorithm is formed. According to the gene sequence, select the selection strategy to combine the factors. The SVM program is used to calculate the individuals in the initial population, find out the predicted value, check whether it corresponds to the sample, calculate the misclassification rate of the test sample, and finally calculate the chromosome individual fitness *F*(*σ*, *C*). Repeat operation *m* times to calculate the fitness value of each individual of the initial population. In order to get the next generation population, crossover, mutation, and selection operators are implemented. The optimal individual is selected and the grid search is carried out near the optimal individual to search the optimal parameter combination. According to the criterion mode of whether the iteration ends or not, if not, continue the iteration and return to recalculate the individual fitness value of chromosome until the conditions are met. Then, the solution of parameter inversion is the optimal individual in the population. The optimal parameter combination, that is, kernel parameters and penalty parameters, is brought into the support vector regression machine program, and a model is created to predict and analyze the data in the sample.

The performance of SVM is determined by the rationality of parameter selection. At present, the conventional method of selecting parameters has great defects, and human factors will lead to the inaccuracy of parameter selection. Using crossover algorithm to select parameters is often accompanied by a huge amount of calculation, and its algorithm is cumbersome. Genetic algorithm has strong search and robust performance and plays a great role in global optimization, which makes the adaptability of genetic algorithm very strong and is widely used in various fields.

After determining the basic data, it is necessary to preprocess the data to increase the accuracy of the model and reduce the prediction error, make the network training faster, and avoid the occurrence of over training. Data normalization is the main work of preprocessing. This operation is to control the data within a certain range by using a certain function or method, such as converting all the data into 0 ∼ 1. This operation is mainly to eliminate the differences between training data. In MATLAB, there are the following normalization methods: first, postmnmx, premnmx, mapminmax, tramnmx, and scaleforsvm; second, trastd, poststd, prestd. The third is programming in MATLAB. There are two programming algorithms, namely, the maximum and minimum method and the mean variance method [19]. For the error measurement in the data, this study adopts the root mean square error and relative error analysis method, where RMSE represents the root mean square error and RE represents the relative error [20].

By preprocessing the influencing factors, the GA-SVM algorithm is empirically analyzed and compared with the traditional SVM algorithm. The results show that the application effect of GA-SVM is obviously better than cross validation SVM, and it is more suitable for precision prediction.

## 4. Experimental Design and Analysis

This study uses the financial data of 132 enterprises in a region in 2019, excluding 30 abnormal data, and the number of effective data samples is 102. The following factors are selected as influencing factors, including accounts receivable turnover rate, operating cash to total debt ratio, return on net assets, quick ratio, current ratio, cost profit ratio, working capital to total assets ratio, main business profit ratio, total asset turnover rate, etc., and the data affecting enterprise financial information processing from 2010 to 2018 are extracted [21, 22].

**Figure 4:**
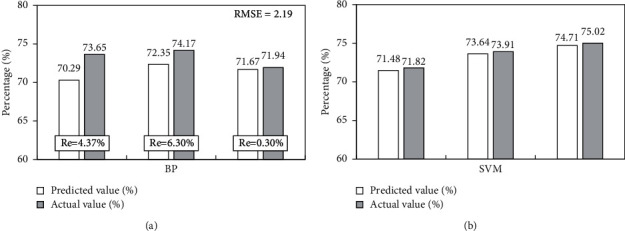
Prediction results and error analysis of BP neural network and SVM. (a) BP neural network. (b) Support vector machine.

Firstly, the results and errors of BP neural network prediction are analyzed. It can be seen that the errors are less than 10%, which meets the prediction requirements, as shown in Figure 4(a) [23, 24]. In the analysis of SVM prediction model, the first eight influence factors are used as the input vector, and the ninth influence factor is used as the output vector to establish the SVM model, and the simulation test is carried out on MATLAB [9,25]. The Gaussian radial kernel function is selected as the kernel function predicted in this study, and the parameters of SVM are selected by cross validation. The parameters are *σ*=0.02, *g*=0.105, *C* = 256. The prediction results are shown in Figure 4(b).

**Figure 5:**
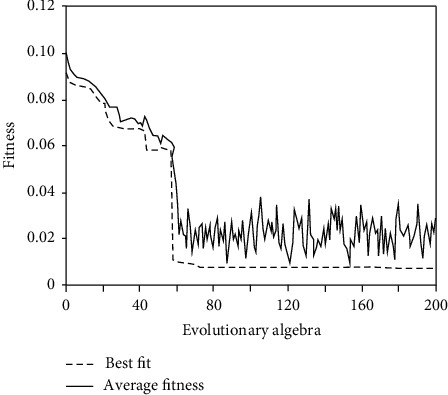
Fitness curve of genetic algorithm optimization.

Figure 4(b) shows the prediction results of SVM. Next, this method will be compared with GA-SVM prediction model. For the selection of prediction data of GA-SVM model, the data from 2010 to 2014 are selected as training samples and the data from 2015 to 2019 are selected as test samples to obtain the fitness of optimization parameters of GA, as shown in Figure 5.

The data from 2010 to 2014 are selected as training samples and the data from 2015 to 2019 are selected as test samples to obtain the fitness of GA optimization parameters, as shown in Figure 5. As can be seen from Figure 5, the average fitness is very close to the best fitness, indicating good results. Comparing the prediction results of SVM model and GA-SVM model, the fitting value selects the data from 2010 to 2014, and the prediction value selects the data from 2015 to 2019. The results show that the prediction errors of SVM model and GA-SVM model are very small, both less than 1%, which meet the prediction requirements. The prediction results of SVM model and GA-SVM model are better than BP neural network.

**Figure 6:**
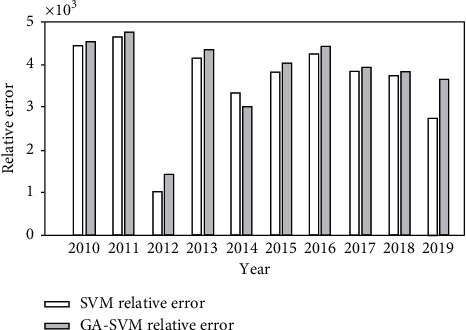
Error effect comparison between GA-SVM and SVM.

The RMSE of GA-SVM is 0.35, and the root mean square error is greater than this value, which shows that the application effect of GA-SVM is obviously better than that of SVM with cross validation method, and it is more suitable for accuracy prediction. At the same time, it also reflects that the genetic algorithm is reliable for the optimization of support vector machine parameters. The error comparison between SVM model and GA-SVM model is shown in Figure 6.

**Figure 7:**
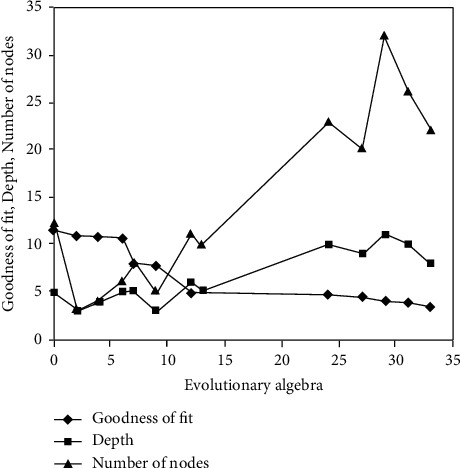
The relationship between fitting degree and tree nodes and layers.

In order to further verify the effectiveness of the model, the factor fat is introduced into the program_ 1, FAT_ 2, and FAT_ 3, the functional relationship between financial security and various factors is mined, and the optimal number of nodes, tree structure diagram, variation evolution and crossover probability change diagram, and fitting optimization diagram of individual algorithm are obtained in the calculation process for simulation and empirical analysis. After the program is simulated, the dynamic change trend of levels, nodes, and goodness of fit of the structure tree is obtained, as shown in Figure 7.

**Figure 8:**
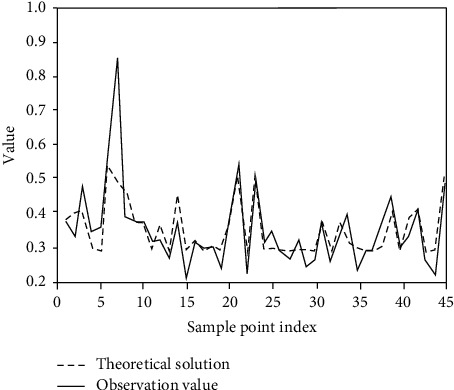
Training sample fitting effect chart.

As can be seen from Figure 7, with the increase of genetic algebra, the goodness of fit will continue to improve. However, after 34 generations, the efficiency of evolution began to remain unchanged, and the current optimal individual was recorded, including the number of nodes of the individual structure and the depth of the tree. FAT of all data samples_ 1, FAT_ 2, FAT_ The value of 3 is introduced into the optimal structural calculation to obtain the observation of enterprise financial standards and the fitting of theoretical values, as shown in Figure 8.

As can be seen from Figure 8, the evaluation model proposed in this study reflects the ups and downs in the process of financial data processing. Using the model to test the sample data, the accuracy of the model is 87.51%. The experimental results fit the financial data well.

By comparing the prediction results of SVM model and GA-SVM model, the fitting value selects the data from 2010 to 2014, and the prediction value selects the data from 2015 to 2019. The results show that the prediction errors of SVM model and GA-SVM model are very small, both less than 1%, which meet the prediction requirements. The prediction results of GA-BP model and SVM-BP model are better than those of SVM-BP model. This shows that the algorithm has a high practical application in enterprises.

## 5. Conclusion

In this paper, a GA-SVM optimization algorithm model based on Internet of Things is established for enterprise financial information management, and the simulation experiment is carried out on MATLAB platform. The results show that the Gaussian radial kernel function is selected as the kernel function predicted in this paper, and the parameters of SVM are selected by cross validation. The parameters are = 0.02, *g*=0.105, *C* = 256. For the selection of prediction data of GA-SVM model, the data from 2010 to 2019 are selected as training samples and the data from 2011 to 2015 are selected as test samples. The value of average fitness of GA-SVM is close to the best fitness, indicating that each individual in the population is near the optimal solution, reflecting the good effect of the algorithm. The RMSE of GA-SVM is 364.062, and the root mean square error obtained is greater than this value, which shows that the application effect of GA-SVM is significantly better than that of SVM using cross validation method, and it is more suitable for precision prediction. At the same time, it also reflects that the optimization of SVM parameters by GA is reliable. This study also has some shortcomings. Due to many influencing factors of enterprise financial management, this study only selects some as the research. In the future research, we will pay more attention to the selection of influencing factors of the model.

## Data Availability

The data used to support the findings of this study are available from the corresponding author upon request.
